# In-vitro effects of the urotensin-II peptide and its receptor antagonists on mcf-7 breast cancer cell line

**DOI:** 10.1186/s43046-026-00366-7

**Published:** 2026-06-13

**Authors:** Murat Olukman, Ezgi Olukman, Kutay Bulut, Burcu Bölük, Sunde Yılmaz Süslüer, Ayşe Erol

**Affiliations:** 1 Faculty of Medicine, Department of Medical Pharmacology, Ege University, Izmir, Turkey; 2 Biomedicine, University of Melbourne, Melbourne, Australia; 3 Faculty of Medicine, Department of Medical Biology, Ege University, Izmir, Turkey

**Keywords:** Urotensin, Breast cancer, Cell culture, Metastasis

## Abstract

**Background:**

Urotensin-II is a powerful vasoconstrictor peptide. Both the peptide and its receptor, GPR14, affect central nervous system, cardiovascular system, and kidney functions. Urotensin-II was found to be involved in the development of many solid organ tumors. The peptide and its receptor have been demonstrated to be present in breast cancer tissue. Moreover, its human plasma level is increased in breast cancer patients. It has also been reported that some cancer patients may have urotensin gene polymorphisms. Antagonists of this peptide receptor with different chemical structures have been produced and studied in different disease models. There are no experimental or clinical studies investigating the effects of urotensin-II receptor antagonists on breast cancer. In this study, MCF-7 cells were grown in an appropriate environment and then plated in wells. WST-8 was used to detect cell proliferation and inhibition effects. The expression levels of the MTA-1, ESR1, Kiss1, UT-II and UTR genes were measured via quantitative RT‒PCR. This study is the first to show the effects of urotensin-II peptide and its receptor antagonists on breast cancer.

**Results:**

While urotensin-II increased cell proliferation, all the antagonists had inhibitory effects. When the expression of genes affecting the metastasis or suppression of tumor cells was investigated, urotensin-II increased MTA-1 and ESR1 expression and decreased Kiss1 expression. Urotensin-II receptor antagonists reversed these effects.

**Conclusions:**

As a result, while urotensin-II triggered breast cancer cell proliferation, its receptor antagonists had inhibitory effects on proliferation and positive regulatory effects on tumor suppressor genes. These results suggest that urotensin-II receptor antagonists may provide effective results for breast cancer treatment.

## Introduction

Urotensin-II (UT-II) is the most potent vasoconstrictor peptide known, exerting its effects through the urotensin-II receptor (UTR, GPR14), a member of the G protein-coupled receptor family [1]. The activation of UTRs in vascular smooth muscle increases inositol triphosphate turnover and intracellular calcium levels, leading to vasoconstriction and various ionotropic effects. However, UT-II can produce opposing effects depending on the species and vascular bed. When UTRs on vascular smooth muscle cells are activated, they induce contraction, whereas UTRs within the endothelium promote vasodilation via nitric oxide signaling. UTR activation triggers a cascade of intracellular signaling events. Specifically, the activation of myosin light-chain kinase and protein kinase promotes vasoconstriction, whereas extracellular signal-regulated kinase, mitogen-activated protein kinase, and p38 mitogen-activated protein kinase B and focal adhesion kinase drive cell proliferation. Additionally, the combined activation of protein kinase B and focal adhesion kinase facilitates cell hypertrophy, adhesion, and invasion [2].

UT-II has been implicated in various chronic diseases. Elevated UT-II levels in humans have been linked to heart failure, renal failure, liver diseases, and diabetes. Moreover, studies suggest a potential association between urotensin and solid organ cancers, including prostate cancer, bladder cancer, hepatocellular carcinoma, colorectal carcinoma, glioblastoma, and breast cancer [2].

Breast cancer is the most common cancer among women and the leading cause of cancer-related deaths, with a concerning increase in its global incidence. Breast cancer can be classified in several ways. Pathologically, it is categorized as either ductal or lobular carcinoma depending on its origin within the breast tissue. Molecular classification, which plays a crucial role in determining treatment strategies, examines the presence or absence of estrogen receptor (ER), progesterone receptor (PR), and human epidermal growth factor receptor 2 (HER2) in cancer cells. The more common type of breast cancer is positive for these receptors. However, triple-negative breast cancer (TNBC), which lacks ER, PR, and HER2 expression, is more prevalent among younger women and has a worse prognosis [3].

Another classification system integrates both pathological and molecular features. Luminal A breast cancer, the most common subtype, is ER(+) and PR(+). Luminal B is also ER(+) and PR(+) but is distinguished by high Ki67 gene expression. The basal-like subtype, also known as TNBC, is characterized by ER(-), PR(-), and HER2(-) [3].

Despite its high incidence, breast cancer mortality has declined in developed countries because of early detection programs. However, metastasis remains the leading cause of breast cancer-related death, accounting for 90% of cases. The most common metastatic sites, in order of frequency, are the bones, lungs, liver, and brain. Tumor suppressor genes play a critical role in reducing the risk of metastasis. Genes such as kisspeptin (Kiss1), E-cadherin, Nm23, TIMPs, and Maspin have been associated with a lower likelihood of tumor spread, whereas genes such as ESR1 and MTA1 are linked to increased metastasis [4].

The relationship between breast cancer and the UT-II peptide has not been well studied, with only a few publications addressing this topic. One study immunohistochemically examined the presence of UT-II and its receptor (UTR) in tumor tissues from 59 breast cancer patients and demonstrated a strong positive correlation in 53 cases [5]. This study reported no significant association between UT-II positivity and patient age but noted a weak correlation between UTR positivity and patient age. Additionally, both UT-II and UTR positivity were significantly greater in premenopausal patients [5]. Other studies suggest that UTR polymorphisms may have prognostic implications in breast cancer [6].

Currently, there is no experimental or clinical research on UTR antagonists in breast cancer despite the development of several UTR antagonists. The most extensively studied and commercially available options include nonpeptide antagonists such as palosuran and SB657510, as well as the peptide-based antagonist urantide. Among these, palosuran has been investigated in a clinical trial for its effects on renal function in diabetic patients — the only clinical study on UTR antagonists to date. Several newly developed antagonists exist but are not yet commercially available.

In this study, we examined the effects of the human UT-II peptide and UTR antagonists, Palosuran, SB657510, and Urantide, on the proliferation of the luminal A breast cancer cell line MCF-7 (ER+, PR+, HER2+). The reason we used MCF-7 cell line in this study is that the most common type of breast cancer is invasive ductal carcinoma. Additionally, we investigated their impact on the expression of the Kiss1, MTA1, and ESR1 genes involved in breast cancer metastasis, as well as the effects of UTR antagonists on UT-II and UTR gene expression.

## Materials and methods

### Replication of the MCF-7 cell line and cell culture studies

Second-passage MCF-7 breast cancer cells were obtained from the Department of Medical Biology (American Type Culture Collection (ATCC)) and cultured in DMEM supplemented with 10% FBS, 1% penicillin/streptomycin, and 1% L-glutamine. The cells grown in the 25 cm^2^ flasks were removed with trypsin upon covering 80–90% of the surface area. The cells were counted and passaged accordingly.

### Cell proliferation and inhibition assays with wst-8

Once a sufficient number of cells were obtained, 5000 cells per well were seeded into 96-well plates and allowed to adhere for 24 h. UT-II was added to the wells at concentrations ranging from 1 × 10⁻⁵ to 1 × 10⁻^10^ M. After 24 and 48 h of incubation, 10 µL of WST-8 was added to each well, and the absorbance was measured spectrophotometrically (450/620 nm) at 30, 60, 120, 180, and 240 min. The results were compared with those of a control group. The same protocol was used for the UTR antagonists palosuran, SB657510, and urantide.

### Real-time PCR studies

For PCR studies, 1 × 10⁵ cells per well were seeded into 6-well plates and allowed to adhere for 24 h. UT-II and UTR antagonists were added at concentrations that demonstrated the most significant effects on proliferation or inhibition in the WST assays. After 24 and 48 h of incubation, the cells were harvested with trypsin, and total RNA was isolated based on the IC_50_ values via LightCycler 480 (Roche) according to the kit manual. The isolated total RNA was transferred to cryotubes and stored at -86 °C until cDNA synthesis. The concentration and purity of the total RNA were measured from 1 to 1.5 µL samples with NanoDrop (Thermo Scientific) at 260/280 nm and 230/260 nm. Samples with a total RNA value greater than 2 were used for further gene expression profile analysis. cDNA synthesis from high-quality RNA was performed, which served as a template for qRT‒PCR. The gene expression levels were determined via the TaqMan method. The abovementioned studies were performed using LightCycler 480 (Roche). Standard series and melting curve analyses were conducted in four logarithmic phases to confirm the efficiency of qRT‒PCR. Gene expression profiles were normalized via the 2^−ΔΔCT^ method. Categorical variables were expressed as frequencies and compared using the χ² test. One-way ANOVA was conducted for statistical analysis, followed by Tukey’s post hoc test for intragroup comparisons. All primers used are listed in Table 1. All the graphs and statistics were generated via GraphPad Prism 8.0.

**Table 1:** List of primers

Genes	Forward	Reverse
UT-II	GCCACTTCAACTCATATCCAAGC	CTCTGGCAGTATCTGTAGAAGGG
UTR	CCCCAACGCAACCCTCAACA	GGTCGCGGTAGTTCCTGGTGA
MTA1	CCAGGACCAAACCGCAGTAACA	GTCAGCTTCGTCGTGTGCAGAT
ESR1	GCTTACTGACCAACCTGGCAGA	GGATCTCTAGCCAGGCACATTC
Kiss1	TTCTAGACCCACAGGCCAGCA	GACGGCTCAGCCTGGCAGT
GAPDH	GTCTCCTCTGACTTCAACAGCG	ACCACCCTGTTGCTGTAGCCAA

## Results

### Cell proliferation and inhibition

**Fig. 1:**
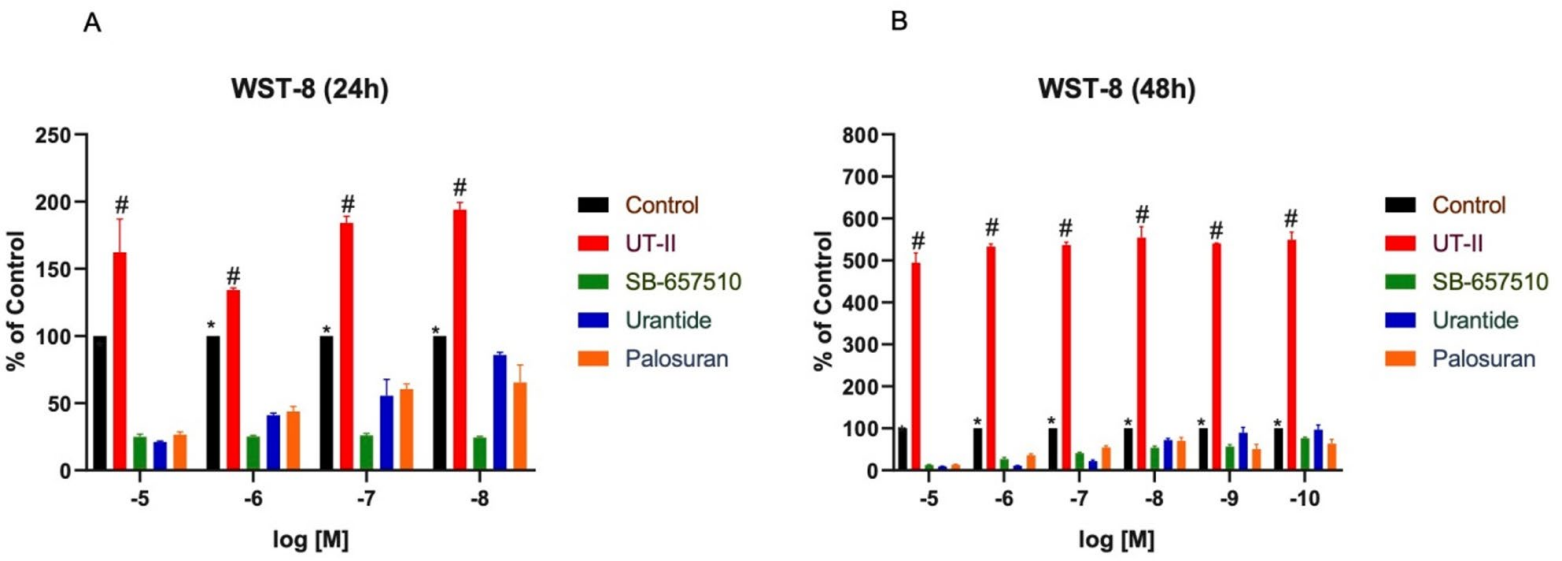
Effects of UT-II and its receptor antagonists on MCF7 cell growth. The control cells were treated with 0.1% DMSO (vehicle). All groups were statistically significant compared to the control (* *p* < 0.01, *n* = 3) and UT-II (# *p* < 0.0001, *n* = 3). The data were analyzed via one-way analysis of variance and Tukey’s post hoc tests. The results are displayed as the means ± standard deviations (SDs). **A** The UT-II peptide promoted cell proliferation after 24 h of incubation. All the antagonists inhibited cell proliferation in a dose-dependent manner. (Control vs. UT-II * *p* = 0.0009; Control vs. SB657510 * *p* = 0.0004; Control vs. Urantide * *p* = 0.0154; Control vs. Palosuran * *p* = 0.0118; UT-II vs. all antagonists # *p* < 0.0001; *n* = 3). **B** UT-II treatment led to a significant increase in cell viability, whereas SB657510, urantide and palosuran treatment resulted in a decrease in cell viability after 48 h of incubation. (Control vs. UT-II * *p* < 0.000; Control vs. SB657510 * *p* = 0.0055; Control vs. Urantide * *p* = 0.0141; Control vs. Palosuran * *p* = 0.0096; UT-II vs. all antagonists # *p* < 0.0001; *n* = 3)

As shown in Fig. 1A and B, the UT-II peptide increased cell proliferation in a dose-dependent manner at both 24 and 48 h of incubation. When applied separately, all antagonists significantly reduced this effect in a dose-dependent manner at both time points. Among them, SB657510 exhibited the strongest inhibitory effect. All the results were statistically significant.

### Gene expression

Following WST-8 analysis, the IC_50_ for each antagonist was determined. The UT-II peptide and UTR antagonists at these concentrations were added to 6-well cell culture plates. The cells were harvested at 24 and 48 h, followed by RNA extraction and cDNA synthesis. The expression levels of the MTA1, ESR1, and Kiss1 genes, as well as the UT-II and UTR genes were quantified via specific gene primers. All the results were normalized to those of the control group.

#### MTA-1

**Fig. 2:**
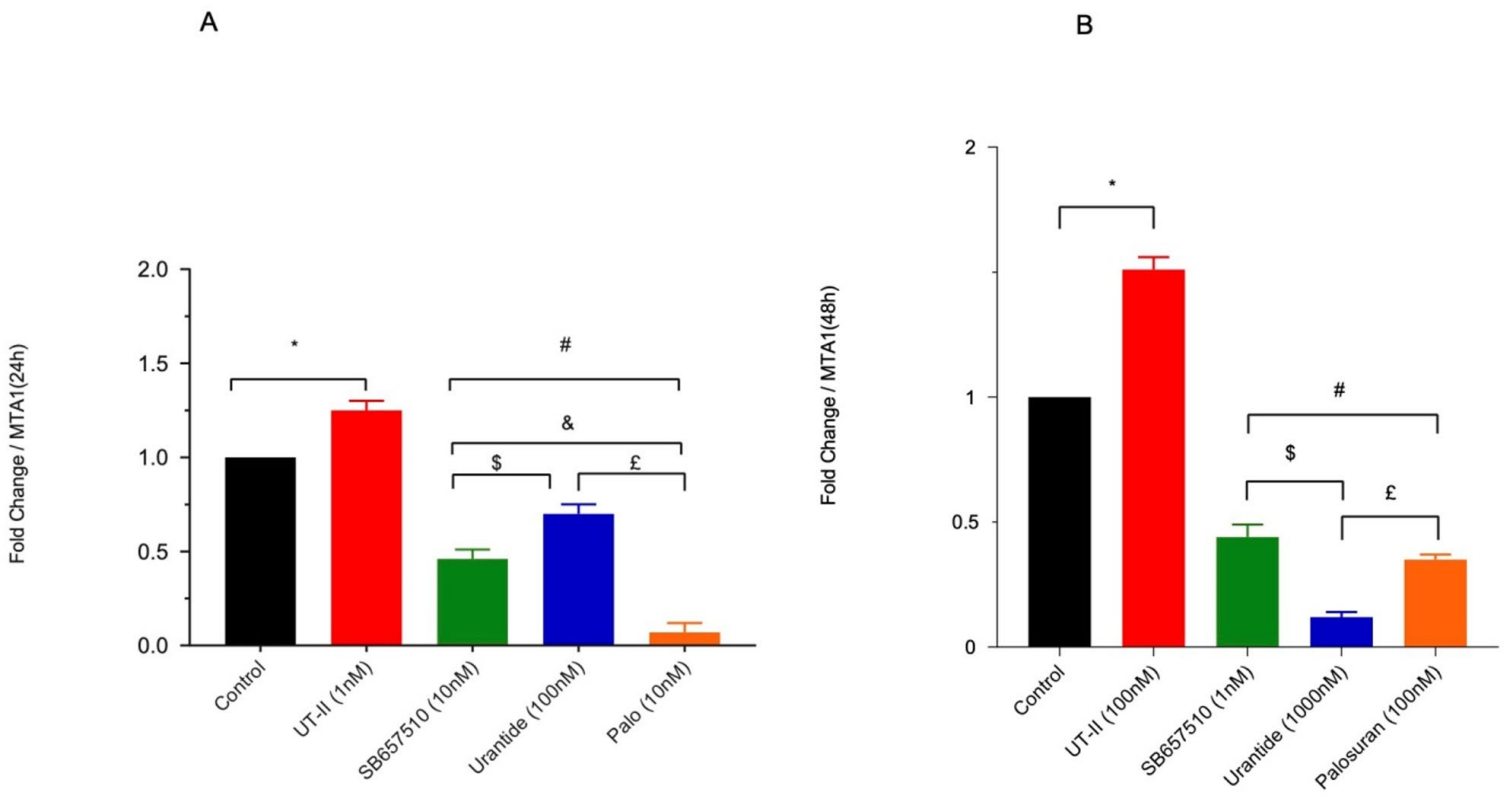
Quantitative real-time PCR was performed to investigate the mRNA expression levels of MTA-1 in the MCF-7 cell line treated with UT-II, SB657510, urantide, and palosuran. The data were analyzed via one-way analysis of variance and Tukey’s post hoc tests. The results are displayed as the means ± standard deviations (SDs). **A** After 24 h of incubation, the expression of MTA-1 was significantly greater in the UT-II group than in the control group and significantly lower in the SB657510, urrantide, and palosuran groups than in the control and UT-II groups. (Control vs. UT-II * *p* = 0.0003; Control vs. all antagonists # *p* < 0.0001, SB657510 vs. Urantide $ *p* = 0.0005; SB657510 vs. Palosuran & *p* < 0.0001; Urantide vs. Palosuran £ *p* < 0.0001; *n* = 3). **B** After 48 h of incubation, the expression of MTA-1 was significantly greater in the UT-II group than in the control group and significantly lower in the SB657510, urrantide, and palosuran groups than in the control and UT-II groups. (Control vs. UT-II * *p* = 0.0001; Control vs. all antagonists # *p* < 0.0001, SB657510 vs. Urantide $ *p* < 0.0001; Urantide vs. Palosuran £ *p* < 0.0001; *n* = 3)

PCR analysis of cDNA from MCF-7 cells exposed to UT-II and UTR antagonists for 24 and 48 h revealed that UT-II significantly upregulated MTA1 gene expression, whereas all the antagonists downregulated it. These results were statistically significant (Fig. 2A and B).

#### ESR1

**Fig. 3:**
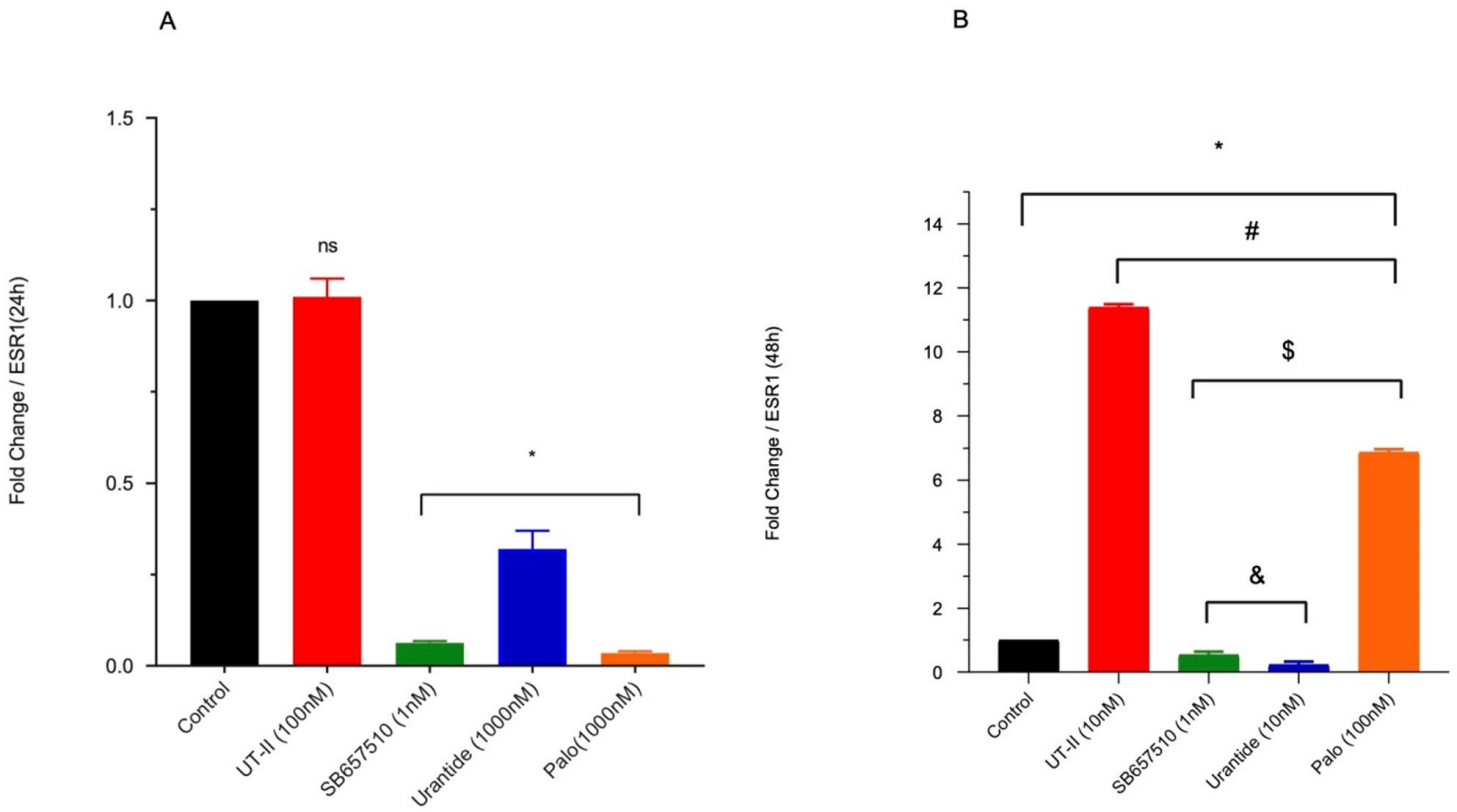
Quantitative real-time PCR was performed to investigate the mRNA expression levels of ESR-1 in the MCF-7 cell line treated with UT-II, SB657510, urrantide, and palosuran. The data were analyzed via one-way analysis of variance and Tukey’s post hoc tests. The results are displayed as the means ± standard deviations (SDs). **A** After 24 h of incubation, the UT-II peptide had no significant effect on ESR1 gene expression compared to the control, whereas all antagonists reduced ESR1 expression compared with both the control and UT-II-treated groups. (Control vs. UT-II ns: not significant; Control vs. all antagonists * *p* < 0.0001; *n* = 3). **B** UT-II had no effect on ESR1 expression during the first 24 h but significantly increased ESR1 expression at 48 h compared with that of control. While SB657510 and urantide decreased ESR1 expression, palosuran significantly increased ESR1 expression, although to a lesser extent than UT-II did. (Control vs. all groups * *p* < 0.001; UT-II vs. all antagonists # *p* < 0.0001; SB657510 vs. Palosuran $ *p* < 0.0001; SB657510 vs. Urantide & *p* = 0.0115; *n* = 3)

PCR analysis of cDNA from MCF-7 cells exposed to UT-II and UTR antagonists for 24 h revealed that UT-II had no significant effect on ESR1 gene expression, whereas all the antagonists significantly decreased it (Fig. 3A). However, 48 h of incubation demonstrated that UT-II significantly decreased ESR1 gene expression. While SB657510 and urantide signficiantly decreased ESR1 expression, palosuran significantly increased it (Fig. 3B).

#### Kiss1

**Fig. 4:**
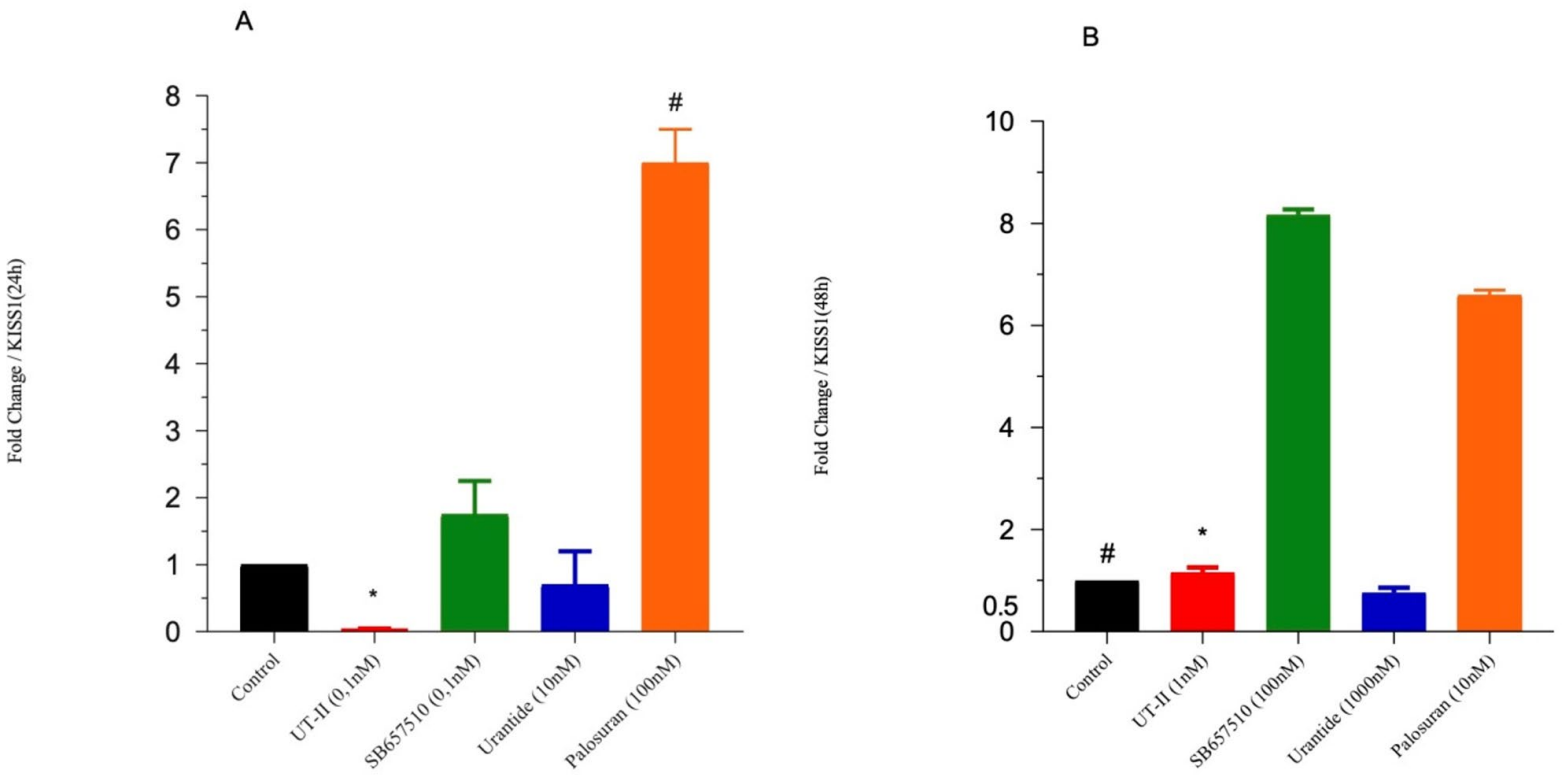
Quantitative real-time PCR was performed to investigate the mRNA expression levels of Kiss1 in the MCF-7 cell line treated with UT-II, SB657510, urrantide and palosuran. The data were analyzed via one-way analysis of variance and Tukey’s post hoc tests. The results are displayed as the means ± standard deviations (SDs). **A** After 24 h of incubation, UT-II significantly decreased Kiss1 expression. While SB657510 and urantide did not significantly increase Kiss1 expression compared to the control, SB6571510 showed significant increase compared to UT-II group. In contrast, palosuran significantly increased Kiss1 expression compared to both UT-II and the control groups. (Control vs. UT-II * *p* < 0.0154; UT-II vs. SB657510 * *p* = 0.0021; UT-II vs. Palosuran * *p* < 0.0001; Palosuran vs. Control, UT-II, SB657510, Urantide # *p* < 0.0001; *n* = 3). **B** After 48 h of incubation, the previous effect of UT-II was reversed, with no significant difference from that of the control. Compared with the control and UT-II groups, SB657510 and palosuran increased Kiss1 expression, whereas urantide had no effect. (Control vs. SB657510, Palosuran # *p* < 0.0001; UT-II vs. SB657510, Palosuran *p* < 0.0001; *n* = 3)

Kiss1 gene expression revealed that UT-II significantly decreased its expression at 24 h. While SB657510 and urantide did not significantly increase Kiss1 expression compared to the control, SB657510 showed significant increase compared to UT-II group. In contrast, palosuran significantly increased Kiss1 expression compared to both UT-II and the control groups (Fig. 4A). After 48 h of incubation, the previous effect of UT-II was reversed, with no significant difference from that of the control. Compared with the control and UT-II groups, SB657510 and palosuran increased Kiss1 expression, whereas urantide had no effect (Fig. 4B).

### UT-II and UTR gene expression PCR results

**Fig. 5:**
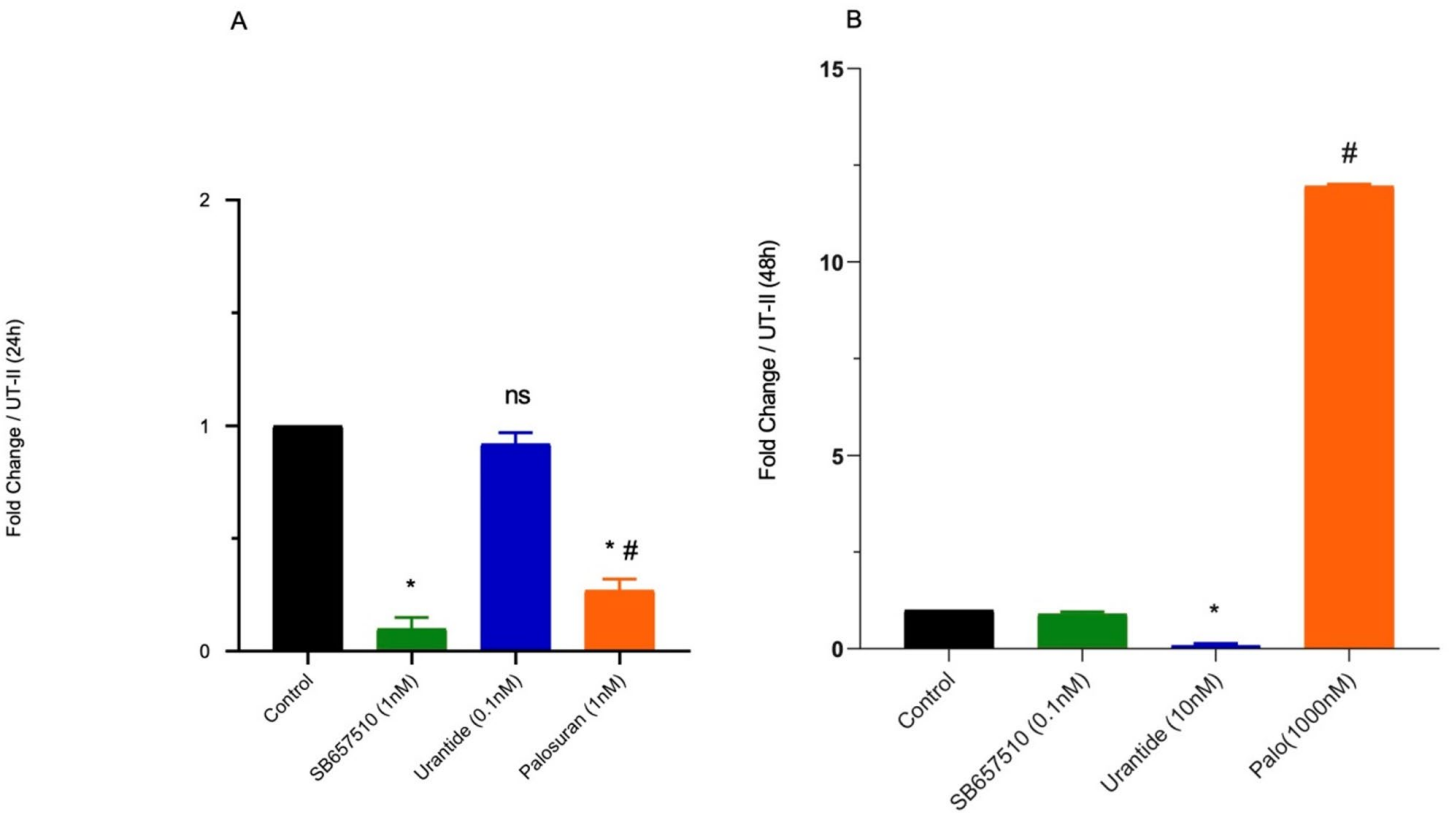
Quantitative real-time PCR was performed to investigate the mRNA expression levels of UT-II in the MCF-7 cell line treated with SB657510, urantide or palosuran. The data were analyzed via one-way analysis of variance and Tukey’s post hoc tests. The results are displayed as the means ± standard deviations (SDs). **A** After 24 h of incubation, urantide had no significant effect on UT-II peptide gene expression, whereas both SB657510 and palosuran reduced its expression. (Control vs. Urantide ns: not significant; Control vs. SB657510, Palosuran * *p* < 0.0001; SB657510 vs. Palosuran # *p* = 0.0059; *n* = 3). **B** After 48 h of incubation only urantide significantly decreased UT-II peptide gene expression. SB657510 did not significantly change, but palosuran significantly increased its expression. (Control vs. Urantide * *p* < 0.0001; Palosuran vs. Control, SB657510, Urantide # *p* < 0.0001; *n* = 3)

**Fig. 6:**
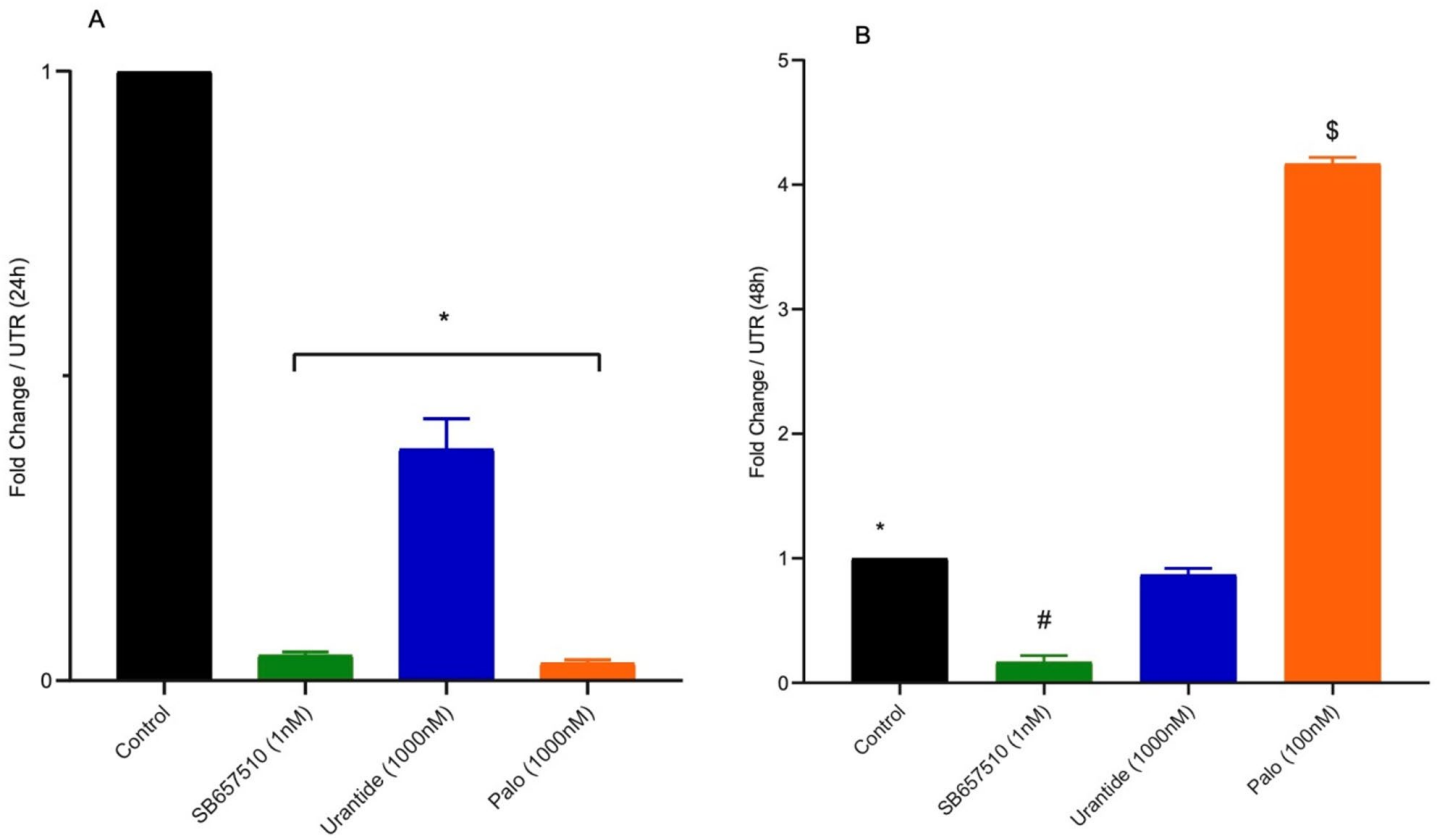
Quantitative real-time PCR was performed to investigate the mRNA expression levels of UTR in MCF-7 cells treated with SB657510, urantide or palosuran. The data were analyzed via one-way analysis of variance and Tukey’s post hoc tests. The results are displayed as the means ± standard deviations (SDs). **A** All the antagonists significantly reduced UTR gene expression at 24 h. (Control vs. all antagonists * *p* < 0.0001; *n* = 3). **B** All the antagonists significantly reduced UTR gene expression at 24 h. (Control vs. all antagonists * *p* < 0.0001; *n* = 3)

Urantide had no significant effect on UT-II peptide gene expression at 24 h, whereas both SB657510 and palosuran reduced its expression (Fig. 5A). However, after 48 h, only urantide significantly decreased UT-II peptide gene expression. SB657510 did not significantly change, but palosuran significantly increased its expression (Fig. 5B). All the antagonists significantly reduced UTR gene expression at 24 h (Fig. 6A). After 48 h, SB657510 and urantide significantly reduced UTR gene expression, whereas palosuran significantly increased its expression (Fig. 6B).

## Discussion

Breast cancer is the most commonly diagnosed cancer in women worldwide, but its mortality rate is decreasing due to the emergence of novel biomarker detection and development of medical devices for early diagnosis. Additionally, advancements in immunotherapy, alongside traditional chemotherapeutics, have reduced the incidence of metastatic tumors [7]. Treatment strategies are now shaped by the identification of molecular subtypes of breast cancer, as the incidence of distant organ metastases varies among subtypes. For example, bone metastases are more common in luminal A breast cancer, whereas lung, brain, and liver metastases are more common in triple-negative breast cancer [3].

Many breast cancer cell lines are used in experimental studies. In our study, we used the MCF-7 cell line, which is classified as Luminal A, because it is the most common breast cancer cell line for this subtype and is positive for ER and PR [8, 9]. Previous research has shown no correlation between UT-II or UTR expression and the molecular structures of breast cancer biopsies. However, UT-II and UTR levels were found to be significantly higher in cancer tissues than in normal breast tissues [10]. Some studies also suggest a potential correlation between UT-II genetic polymorphisms and breast cancer. UT-II has been shown to have increased expression in solid organ cancers such as glioblastoma, neuroblastoma, choriocarcinoma, adrenocortical, and colorectal cancers [11].

Our study demonstrated, for the first time, that UT-II increases MCF-7 cell proliferation in a dose-dependent manner and that UTR antagonists inhibit this proliferation in a dose- and time-dependent manner. UT-II is a peptide whose plasma level increases in conditions such as hypertension, diabetes, and coronary failure. At the cellular level, UT-II is known to promote smooth muscle proliferation and migration primarily through the Rho/Rho-kinase pathway. Additionally, it has been shown that this peptide is a nonclassical angiogenic factor [12]. Given these findings, UT-II may play a role in metastasis; therefore, UTR antagonists could have potential applications in preventing cancer metastasis and invasion.

Urantide, the UTR antagonist in the peptide form used in our study, has previously been shown to reduce the expression of focal adhesion kinase and cell adhesion molecules (CD61 and CD11) in androgen-dependent prostate cancer cells [2]. Additionally, urantide decreased cell invasion and migration by 50% and 35%, respectively, in bladder cancer cells after 48 h of incubation [2]. In our study, urantide significantly decreased cell proliferation at both 24 and 48 h. In the only oncological study conducted on the nonpeptide UTR antagonist SB657510, the antagonist was shown to reduce focal adhesion kinase and protein kinase-induced ERK mitogenic activity in lymphangioleiomyomatosis cells. SB657510 was also shown to reduce ELT-3 tumor development and the circulating tumor cell load in a mouse xenograft model [11]. There are no studies on the UTR antagonist palosuran in the current literature. In our study, all three antagonists decreased MCF-7 cell proliferation at 24 and 48 h (Fig. 1A and B).

Metastasis is the leading cause of breast cancer mortality, and its frequency is partially influenced by genetics. While some genes suppress tumor formation, others promote metastasis. MTA-1 is a metastasis-associated gene that can be upregulated under stress-related conditions, such as hypoxia, radiation, or heat shock [13]. It also plays a role in estrogen receptor signaling. Compared with normal breast cells, significantly increased MTA-1 expression has been observed in MCF-7 cells under hypoxic conditions [14]. Our results revealed that UT-II increased MTA-1 expression, whereas all three antagonists decreased MTA-1 expression. We observed that their activities differed from each other in a dose- and time-dependent manner (Fig. 2A and B).

ER positivity is critical in hormonal therapy for breast cancer, with tamoxifen being widely used. MCF-7 cells are ESR positive [15]. While two of the receptors are nuclear (ESR1 and ESR2), the other is transmembrane (G protein ER1). The nuclear receptor ESR1 is overexpressed in breast cancer and serves as a prognostic marker for treatment. In our study, UT-II had no significant effect on ESR1 expression at 24 h but significantly increased ESR1 expression at 48 h (Fig. 3A). SB657510 and urantide decreased ESR1 expression (Fig. 3B), whereas palosuran’s effect of reducing ESR1 expression at 24 h diminshied by 48 h. Given that MTA-1 regulates estrogen activity, the abovementioned effects of the antagonists may be linked to their ability to suppress MTA-1 expression [14].

The tumor suppressor gene Kiss1 plays an antimetastatic role in various cancers by binding to kisspeptins [16]. Kiss1 receptor expression is elevated in MCF-7 cells [17, 18]. In our study, UT-II significantly suppressed Kiss1 expression at 24 h, although this effect disappeared at 48 h. In contrast, SB657510 and palosuran increased Kiss1 expression (Fig. 4A and B). These findings provide the first evidence of the impact of UTR antagonists on Kiss1 expression in breast cancer.

UT-II and UTR expression have been detected in both healthy and cancerous breast tissue [19]. However, no previous studies have examined the effects of UTR antagonists on breast cancer or their influence on UT-II and UTR gene expression. Our study demonstrated the effects of UTR antagonists on both the expression of UT-II and UTR genes in MCF-7 breast cancer cells. SB657510 and urantide suppressed both UT-II and UTR expression in a time-dependent manner, whereas palosuran increased the expression of both genes (Figs. 5AB and 6AB). Urantide and SB657510 act as competitive antagonists, while palosuran is noncompetitive [20]. At concentrations of 0.1 nM–10 µM, urantide has a competitive antagonistic effect on UT-II in the rat aorta, while palosuran has demonstrated an antagonistic effect at a concentration of K_i_ 5 nM in various disease models [20]. There is no clear information regarding the partial agonist effects of these antagonists. The notable difference in UTR expression at 24 and 48 h following palosuran exposure suggests a potential partial agonist effect that has not previously been reported. We plan to investigate this further using gene silencing techniques targeting the UTR gene.

In conclusion, breast cancer treatment varies by molecular subtype. Hormone receptor-positive cancers are typically treated with ER-targeted therapies and chemotherapeutics. The role of UT-II in breast cancer metastasis remains underexplored. Although UT-II is a known vasoconstrictor, its clinical significance in breast cancer is unclear. Palosuran, the only clinically studied UTR antagonist, has shown efficacy in diabetic nephropathy but has not progressed beyond phase 1 trials. It is necessary to further investigate the impacts of UT-II on breast cancer. In particular, further studies focusing on gene silencing techniques need to be conducted to determine whether these antagonists exert their effects solely through UTR antagonism or by regulating other receptors. Our study demonstrated for the first time that UT-II promotes MCF-7 cell proliferation, whereas UTR antagonists (urrantide, SB657510, and palosuran) inhibit proliferation. Furthermore, we showed that UT-II increases the expression of the metastasis-associated gene MTA-1 and the breast cancer-associated gene ESR1, whereas UTR antagonists decrease the expression of these genes. Additionally, we showed that UT-II suppresses Kiss1, while its antagonists increase its expression. Further research, particularly involving gene silencing, is needed to determine whether these effects are mediated primparily through UTR antagonism or other pathways.

## Data Availability

All genes were sequenced from Pubmed central.

## Abbreviations

UT-II: Urotensin-II
UTR: Urotensin-II Receptor
ER: Estrogen Receptor
PR: Progesterone Receptor
HER2: Human epidermal growth factor receptor 2
TNBC: Triple-negative breast cancer
Kiss1: Kisspeptin
MTA1: Metastasis-associated protein 1
Erα, ESR1: Estrogen receptor α
